# HCC-derived exosomes elicit HCC progression and recurrence by epithelial-mesenchymal transition through MAPK/ERK signalling pathway

**DOI:** 10.1038/s41419-018-0534-9

**Published:** 2018-05-03

**Authors:** Lu Chen, Piao Guo, Yuchao He, Ziye Chen, Liwei Chen, Yi Luo, Lisha Qi, Yuanyuan Liu, Qiang Wu, Yunlong Cui, Feng Fang, Xiaofang Zhang, Tianqiang Song, Hua Guo

**Affiliations:** 1 Department of tumor cell biology, Tianjin Medical University Cancer Institute and Hospital, National Clinical Research Center for Cancer, Key Laboratory of Cancer Prevention and Therapy, 300060 Tianjin, China; 2 Department of Hepatobiliary, Tianjin Medical University Cancer Institute and Hospital, National Clinical Research Center for Cancer, Key Laboratory of Cancer Prevention and Therapy, 300060 Tianjin, China; 3 Department of Pathology, Tianjin Medical University Cancer Institute and Hospital, National Clinical Research Center for Cancer, Key Laboratory of Cancer Prevention and Therapy, 300060 Tianjin, China; 4 Department of Genetics, Basic Medical College, Tianjin Medical University, 300070 Tianjin, China; 5 Department of Medical Laboratory, Tianjin Medical University General Hospital, 300052 Tianjin, China

## Abstract

Liver cancer is the second most common cause of cancer-related death worldwide. Approximately 70–90% of primary liver cancers are hepatocellular carcinoma (HCC). Currently, HCC patient prognosis is unsatisfactory due to high metastasis and/or post-surgical recurrence rates. Therefore, new therapeutic methods for inhibiting metastasis and recurrence are urgently needed. Exosomes are small lipid-bilayer vesicles that are implicated in tumour development and metastasis. Rab27a, a small GTPase, regulates exosome secretion by mediating multivesicular endosome docking at the plasma membrane. However, whether Rab27a participates in HCC cell-derived exosome exocytosis is unclear. Epithelial-mesenchymal transition (EMT) frequently initiates metastasis. The role of HCC cell-derived exosomes in EMT remains unknown. We found that exosomes from highly metastatic MHCC97H cells could communicate with low metastatic HCC cells, increasing their migration, chemotaxis and invasion. Rab27a knockdown inhibited MHCC97H-derived exosome secretion, which consequently promoted migration, chemotaxis and invasion in parental MHCC97H cells. Mechanistic studies showed that the biological alterations in HCC cells treated with MHCC97H-derived exosomes or MHCC97H cells with reduced self-derived exosome secretion were caused by inducing EMT via MAPK/ERK signalling. Animal experiments indicated that exosome secretion blockade was associated with enhanced lung and intrahepatic metastasis of parental MHCC97H cells, while ectopic overexpression of Rab27a in MHCC97H cells could rescue this enhancement of metastasis in vivo. Injection of MHCC97H cell-derived exosomes through the tail vein promoted intrahepatic recurrence of HLE tumours in vivo. Clinically, Rab27a was positively associated with serum alpha-fetoprotein (AFP) level, vascular invasion and liver cirrhosis. Our study elucidated the role of exosomes in HCC metastasis and recurrence, suggesting that they are promising therapeutic and prognostic targets for HCC patients.

## Introduction

Liver cancer is a highly fatal disease and the second most common cause of cancer-related death worldwide^1^. Liver cancer is responsible for more than 700,000 deaths every year worldwide, and China alone accounts for 50% of the total deaths^1,2^. Approximately 70–90% of liver cancers occurring worldwide are hepatocellular carcinoma (HCC)^1^. At present, surgical resection is still the primary treatment method for HCC patients. However, the 5-year risk of recurrence after surgery is as high as 70%, and recurrence often occurs within the first 2 years after resection^3^. This early recurrence is frequently caused by tumour invasion and metastasis. Thus, new treatment strategies to control metastasis and recurrence are urgently needed.

Exosomes are small membrane vesicles with a size between 50 and 140 nm. They are secreted by multiple cell types, including cancer cells^4,5^. Exosomes have a cup-shaped morphology or are round vesicles as shown by transmission and cryo-electron microscopy, respectively^6^. Recent evidence indicates that exosomes can mediate intercellular communication and promote tumourigenesis, tumour immune escape and metastasis^7,8^. Rab27a, a member of the Rab GTPases, functions in multivesicular endosome docking in the plasma membrane, thereby regulating exosome release^9^. Secretion of exosomes in a Rab27a-dependent manner has been revealed in melanoma and breast and bladder cancers; abnormal exosome production caused by modulating Rab27a expression can influence tumour growth, tumour metastasis and progression^10–12^. However, whether Rab27a is responsible for exosome release in HCC and the subsequent effect on biological behaviour in HCC cells is still largely unknown.

Epithelial-mesenchymal transition (EMT) is a process in which epithelial cells lose their polarity and cell–cell junctions and acquire a mesenchymal phenotype with increased migratory and invasive abilities^13,14^. EMT activation has been proposed as a vital mechanism for epithelial cancer cells to acquire a malignant phenotype. Recently, the role of exosomes in the EMT programme has been revealed in different types of cancer, including nasopharyngeal cancer, bladder cancer and melanoma^15–17^. However, whether exosomes promote EMT of HCC cells and the underlying mechanisms remain elusive.

In this report, we carried a systematic study of the role of exosomes in HCC invasion, metastasis and recurrence. We explored the changes in malignant features of HLE and Hep3B cells incubated with MHCC97H-derived exosomes, and we studied the role of Rab27a in exosome secretion and the consequent effect on biological functions of MHCC97H cells. The involvement of EMT and the relevant signalling pathways were also investigated. We further assessed the expression pattern of Rab27a in HCC samples and HCC cells, as well as the correlation between Rab27a and clinicopathological characteristics. Animal experiments indicated the influence of exosomes on HCC metastasis and intrahepatic recurrence. Our research revealed that HCC-derived exosomes could mediate EMT and enhance malignancy of HCC cells, suggesting that they may be novel diagnostic markers and targets for prevention of metastasis and recurrence of HCC.

## Results

### Highly metastatic MHCC97H-derived exosomes improve migration, chemotaxis and invasion of low metastatic HCC cells

**Fig. 1:**
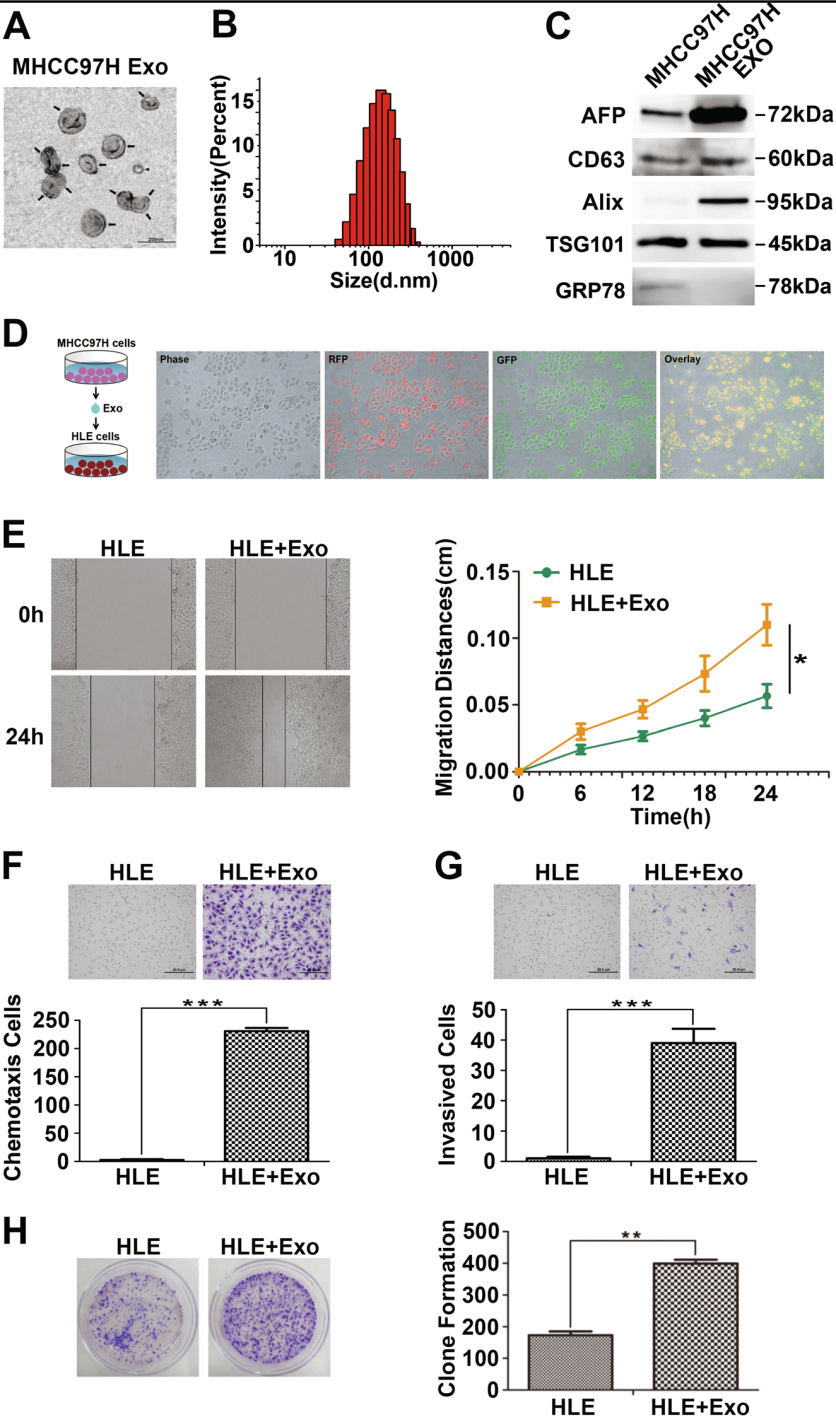
Highly metastatic MHCC97H-derived exosomes improve migration, chemotaxis and invasion of HLE cells. **a** Transmission electron micrograph of MHCC97H exosomes. Arrows indicate exosomes and arrowheads indicate smaller nonexosomal vesicles. Scale bar, 200 nm. **b** The size distribution of isolated exosomes using Zetasizer Nano ZS90. **c** Equal amounts of total protein (60 μg) from MHCC97H cells and exosomes were analysed by Western blot for the presence of the exosomal markers CD63, Alix and TSG101, as well as AFP (a recognised HCC marker). The endoplasmatic reticulum protein GRP78 was used as the negative control. **d** Fluorescence microscopy images of HLE cells treated with fluorescently labelled MHCC97H exosomes (single-stranded RNAs: red; exosomal proteins: green) for 24 h. **e** Scratch assay for the migration of HLE cells treated with or without MHCC97H-derived exosomes (100 μg/ml). The distance was measured every 6 h for 24 h. **f** The chemotactic potential of HLE cells treated with or without MHCC97H-derived exosomes (100 μg/ml). The incubation time was 24 h. **g** Matrigel invasion assay for the invasion of HLE cells treated with or without MHCC97H-derived exosomes (100 μg/ml). The incubation time was 36 h. **h** Colony formation assay of HLE cells treated with or without MHCC97H-derived exosomes (100 μg/ml). The culture time was 2 weeks. Abbreviation: Exo exosome. *P < 0.05, **P < 0.01 and ***P < 0.001. Scale bar, 1.0 mm. Data are represented as the mean ± S.D. All experiments were repeated at least three times

A previous study showed that exosome-mediated transfer of pro-metastatic molecules from malignant cancer cells to less malignant ones can lead to metastatic properties in the recipient cells in breast cancer^18^. Therefore, we investigated whether this phenomenon also existed in HCC cells. First, we used sequential ultracentrifugation to isolate exosomes from the culture supernatant of MHCC97H cells, a highly metastatic HCC cell line^19^. Cup-shaped particles between 50 and 140 nm were identified by transmission electron microscopy (Fig. 1a). The nanoparticle tracking analysis by Zetasizer Nano ZS90 revealed that the average diameter of isolated exosomes was 139 nm (Fig. 1b). Detection of three commonly used exosomal marker proteins, CD63, Alix and TSG101, confirmed the identity of the exosomes (Fig. 1c). Consistent with a previous report^20^, enriched expression of the recognised HCC marker alpha-fetoprotein (AFP) was detected in exosomes compared with the parental MHCC97H cells. To determine the potential of exosomes to be taken up and internalised by other HCC cells, we labelled MHCC97H-derived exosomal RNA with red fluorescence dye and exosomal protein with green fluorescence dye as described in the Materials and methods section. The labelled exosomes were incubated with HLE cells for 24 h, and the internalization was confirmed by fluorescence microscopy (Fig. 1d). Then, we investigated whether MHCC97H-derived exosomes could enhance the tumour-promoting functions of recipient HLE cells. Scratch assays showed a notable increase in the migration of HLE cells when cocultured with MHCC97H-derived exosomes (Fig. 1e). In addition, enhanced chemotaxis and invasion were observed in HLE cells treated with MHCC97H-derived exosomes (Fig. 1f, g). Furthermore, incubation with MHCC97H-derived exosomes induced HLE cells to form more colonies (Fig. 1h), while the increase in proliferation rate was insignificant (Supplementary Figure S1), which might be partly due to the limited growth space and short experiment time compared with clonogenicity assays^21^. We also examined the influence of MHCC97H-derived exosomes on another HCC cell line Hep3B cells, and we got similar results. Hep3B treated with MHCC97H-derived exosomes acquired enhanced capabilities of migration, chemotaxis, invasion and colony formation (Supplementary Figure S2A-D).

Overall, these data suggest that exosomes secreted from highly metastatic MHCC97H cells can enter relatively low metastatic HCC cells, which then significantly enhance the migration, chemotaxis, invasion and colony formation of the recipient HCC cells.

### MHCC97H-derived exosomes induce HCC cells to undergo EMT through the MAPK/ERK pathway

**Fig. 2:**
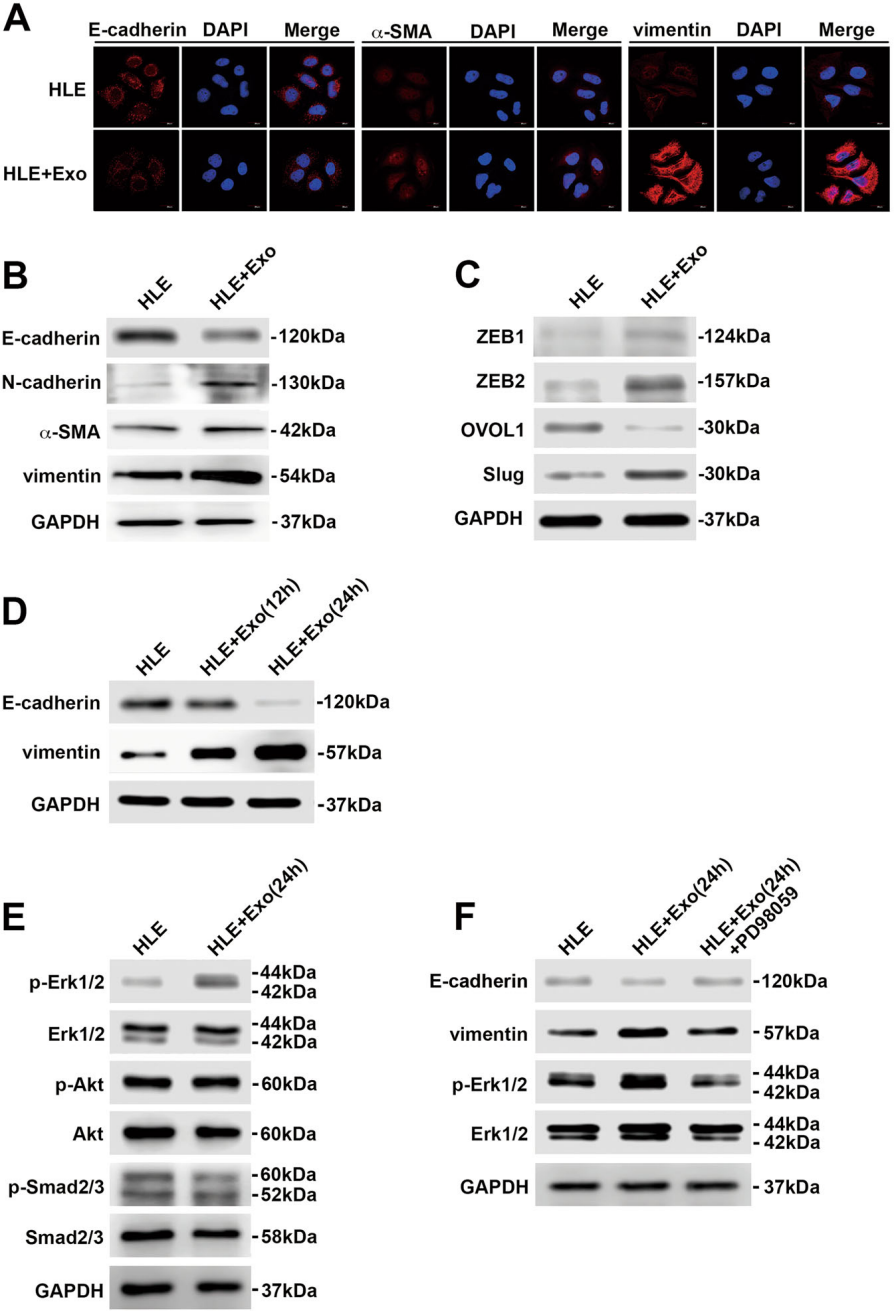
Incubation of HLE cells with MHCC97H-derived exosomes promotes EMT through the MAPK/ERK pathway. **a** Immunofluorescence microscopy of EMT markers in HLE cells treated with or without MHCC97H-derived exosomes for 24 h. **b** Western blot analysis of EMT markers in HLE cells treated with or without MHCC97H-derived exosomes for 24 h. **c** Western blot analysis of EMT promoters (ZEB1, ZEB2 and Slug) and MET promoter OVOL1 in HLE cells treated with or without MHCC97H-derived exosomes for 24 h. **d** Western blot analysis of EMT markers of HLE cells with exposure to MHCC97H-derived exosomes for different durations. **e** Western blot analysis of phosphorylated and total Erk1/2, Akt and Smad2/3 in HLE cells treated with or without MHCC97H-derived exosomes for 24 h. **f** Western blot analysis of EMT markers and phosphorylated and total Erk1/2 in HLE cells, exosome-treated HLE cells and exosome-treated HLE cells when cultured with the ERK inhibitor PD98059. Abbreviation: Exo exosome. All experiments were repeated at least three times

EMT has been implicated in exosome-mediated invasion and metastasis of tumour cells^22^. Thus, we next examined the involvement of EMT and the specific signalling pathways responsible for the functional changes in HLE cells after treatment with MHCC97H-derived exosomes. Immunofluorescence staining showed that higher expression of α-SMA and vimentin (mesenchymal markers) and lower expression of E-cadherin (an epithelial marker) were observed in MHCC97H exosome-treated HLE cells compared to control HLE cells (Fig. 2a). Western blot assays revealed that incubation with MHCC97H-derived exosomes suppressed the expression of the epithelial marker E-cadherin and promoted the expression of mesenchymal markers, such as N-cadherin, α-SMA and vimentin (Fig. 2b). Similar alterations of the expression levels of these EMT markers could be observed in Hep3B cells treated with MHCC97H-derived exosomes (Supplementary Figure S2E). Moreover, an increase in the expression level of EMT promoters (ZEB1, ZEB2 and Slug) and a decrease in that of the MET (mesenchymal-epithelial transition)-driving promoter OVOL1 were observed in HLE cells treated with MHCC97H-derived exosomes compared with control HLE cells (Fig. 2c).

To identify the signalling pathway involved in exosome-mediated EMT, we first examined the expression changes of EMT markers in HLE cells exposed to MHCC97H-derived exosomes (100 μg/ml) for different durations. The results showed that HLE cells treated with MHCC97H-derived exosomes for 24 h appeared to have the highest vimentin levels and the lowest E-cadherin levels (Fig. 2d). As a result, we selected 24 h as the appropriate exposure duration for further analyses. Given the important role of the PI3K/Akt, TGF-β and MAPK/ERK signalling pathways in EMT of HCC^23,24^, we assessed the expression levels of representative proteins in these three pathways. The results showed that phosphorylation of Erk1/2 was significantly elevated in HLE cells incubated with MHCC97H-derived exosomes, while phosphorylation of Akt and Smad2/3 remained equal to that of the control group (Fig. 2e). Then, we applied the ERK inhibitor PD98059 to HLE cells for 1 h prior to MHCC97H-derived exosome treatment. Western blot assays showed that PD98059 conspicuously attenuated the elevated ERK activity, as well as the increased expression of vimentin and the decreased expression of E-cadherin in HLE cells treated with MHCC97H-derived exosomes (Fig. 2f).

In summary, these studies reveal that uptake of MHCC97H-derived exosomes can induce HCC cells to undergo EMT through the MAPK/ERK signalling pathway.

### Rab27a downregulation inhibits MHCC97H-derived exosome secretion and promotes migration, chemotaxis and invasion of parental cells

**Fig. 3:**
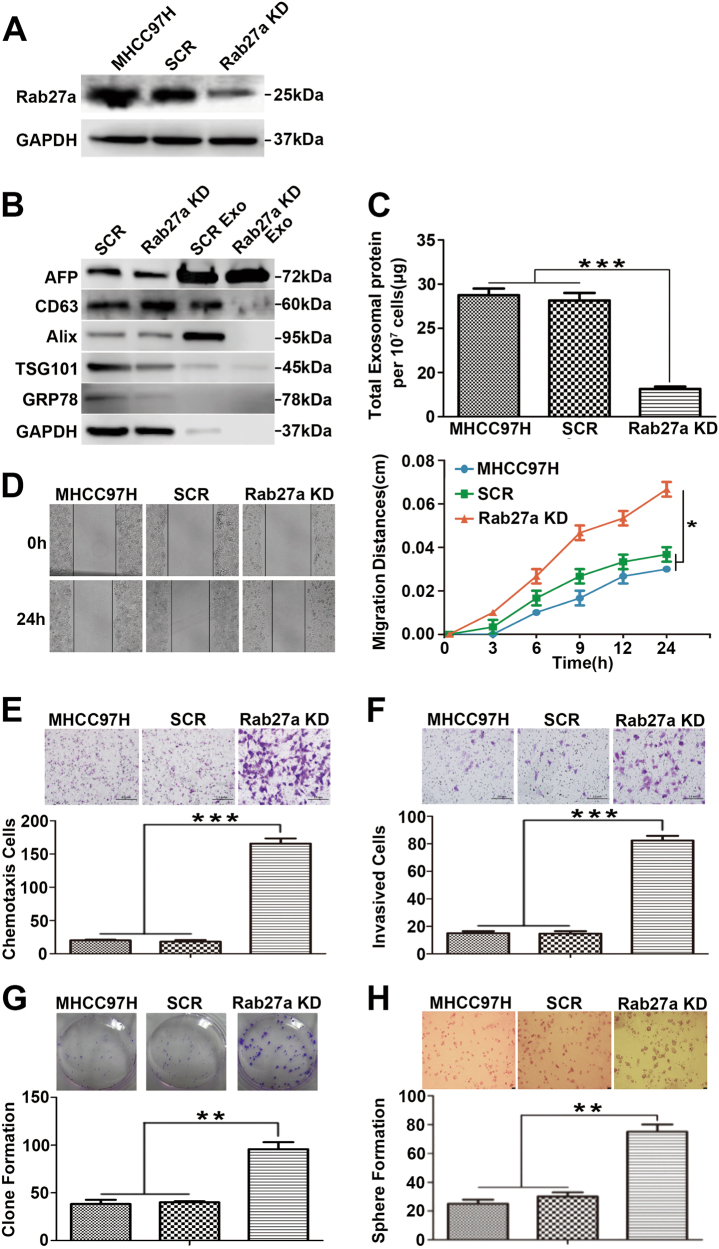
Rab27a downregulation inhibits MHCC97H-derived exosome secretion and promotes migration, chemotaxis and invasion of parental cells. **a** The knockdown efficiency of the Rab27a shRNA was examined by Western blot. **b** Equal amounts of total exosomal proteins (60 μg) secreted by siRab27a/MHCC97H and siSCR/MHCC97H cells were analysed by Western blot for the presence of the exosomal markers CD63, Alix and TSG101, as well as AFP (a recognised HCC marker). The endoplasmatic reticulum protein GRP78 was used as the negative control. GAPDH was used as loading control. **c** The total amounts of protein in exosome pellets purified from culture supernatant of MHCC97H (WT), siSCR/MHCC97H and siRab27a/MHCC97H cells were quantified by Bradford assay. **d** Alteration in migration by Rab27a knockdown was determined by scratch assays. **e** Alteration in chemotaxis by Rab27a knockdown was measured by chemotaxis assays. **f** Alteration in invasion by Rab27a knockdown was examined by Matrigel invasion assays. **g** Alteration in clonogenic ability by Rab27a knockdown was detected by colony formation assays. **h** Alteration in sphere formation ability by Rab27a knockdown was explored by 3D sphere formation assays. Abbreviation: Exo exosome. *P < 0.05, **P < 0.01 and ***P < 0.001. Scale bar, 1.0 mm. Data are represented as the mean ± S.D. All experiments were repeated at least three times

The above results supported the hypothesis that exosomes contained pro-metastatic molecules that promote tumour progression of the recipient cells^25^. Then, we explored the biological effects on parental cells after abrogating the release of these exosomal factors through inhibition of Rab27a-dependent exosome secretion^26^. Initially, we established a stable siRab27a/MHCC97H cell line using a lentivirus expressing shRNA. Western blot analysis demonstrated the efficiency of Rab27a knockdown (Fig. 3a). Then, we examined the effect of Rab27a downregulation on exosome secretion. A reduction in the signals of three exosome markers (CD63, Alix and TSG101) was observed by Western blot analysis of exosomes purified from the culture supernatant of siRab27a/MHCC97H cells compared with control cells (Fig. 3b). Moreover, the total levels of secreted exosomes were significantly decreased in siRab27a/MHCC97H cells compared to control cells (Fig. 3c). Next, we explored the effects of Rab27a knockdown on migration, chemotaxis, invasion and colony formation capacities of MHCC97H cells. Scratch assays showed that the migratory activity of siRab27a/MHCC97H cells was significantly higher than that of control cells (Fig. 3d). Elevated chemotactic movement was found in siRab27a/MHCC97H cells compared with control cells (Fig. 3e). A similar enhancement was observed when assessing invasion of siRab27a/MHCC97H cells (Fig. 3f). In addition, colony formation assays showed that siRab27a/MHCC97H cells had the potential to form more colonies than control cells (Fig. 3g), while Rab27a knockdown did not cause a significant change in the proliferation rate of MHCC97H cells (Supplementary Figure S3). Similarly, 3D sphere formation assays demonstrated that siRab27a/MHCC97H cells formed more spheres than control cells under serum-free condition (Fig. 3h).

Altogether, the data indicate that Rab27a knockdown substantially inhibits exosome secretion, which subsequently promotes migration, chemotaxis, and invasion as well as colony and sphere formation of parental MHCC97H cells.

### Inhibition of self-derived exosome secretion by Rab27a blockade facilitates EMT through the MAPK/ERK pathway

**Fig. 4:**
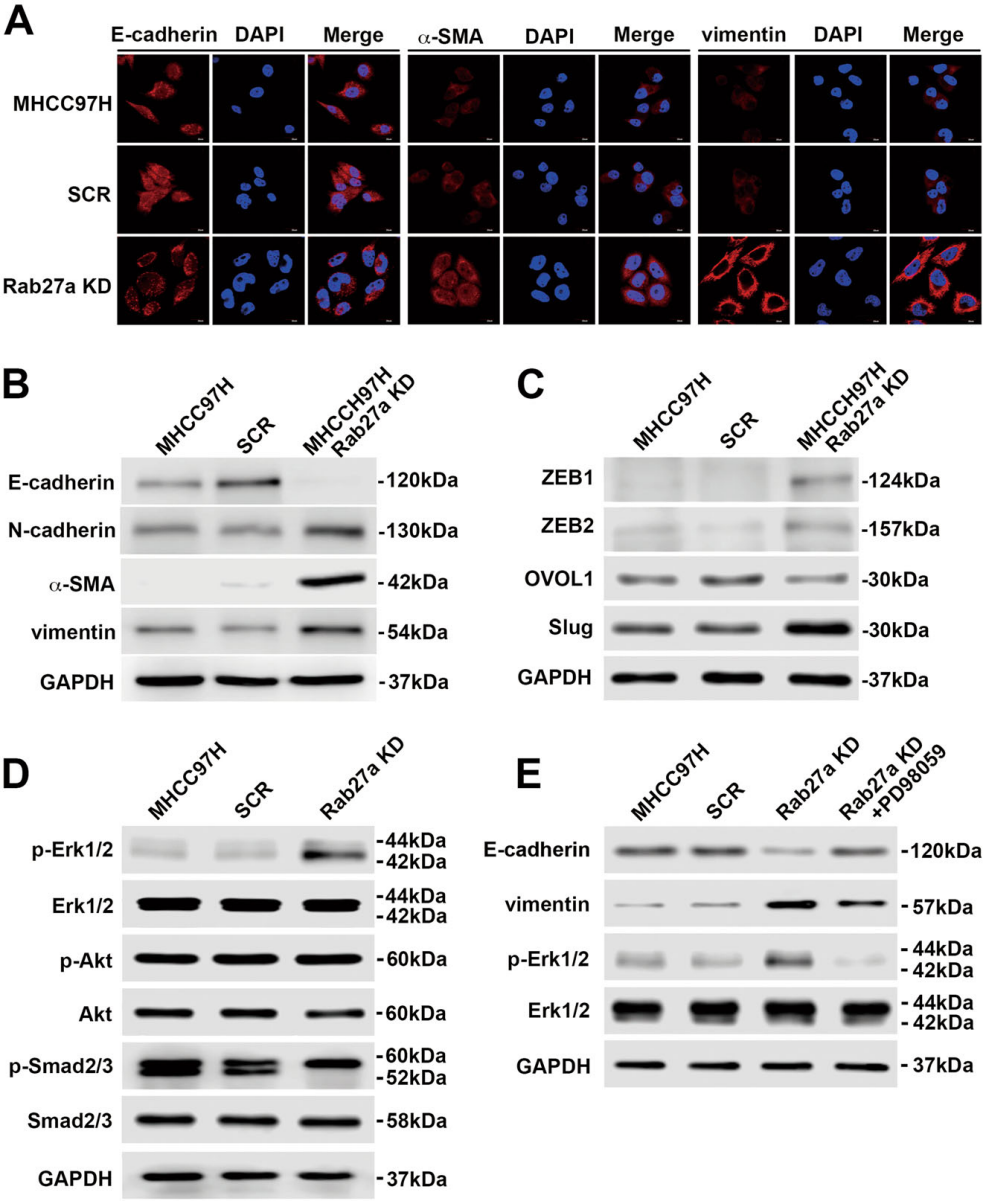
Inhibition of self-derived exosome secretion by Rab27a blockade induces EMT through the MAPK/ERK pathway. Analysis of the expression level of EMT markers in MHCC97H (WT), siSCR/MHCC97H and siRab27a/MHCC97H cells by immunofluorescence microscopy (**a**) and Western blot (**b**). **c** Analysis of the expression pattern of EMT-associated and MET-associated transcriptional regulators in MHCC97H (WT), siSCR/MHCC97H and siRab27a/MHCC97H cells at the protein level. **d** Western blot analysis of phosphorylated and total Erk1/2, Akt and Smad2/3 in MHCC97H (WT), siSCR/MHCC97H and siRab27a/MHCC97H cells. **e** Western blot analysis of phosphorylated and total Erk1/2 and EMT markers in MHCC97H (WT), siSCR/MHCC97H, and siRab27a/MHCC97H cells treated with or without the ERK inhibitor PD98059 for 24 h. All experiments were repeated at least three times

The above studies confirmed that attenuated exosome secretion by Rab27a knockdown enhanced malignant properties of donor MHCC97H cells. We next examined the involvement of EMT in these intrinsic functional alterations in MHCC97H cells after inhibition of exosome secretion. Immunofluorescence staining and Western blot assays showed that Rab27a knockdown increased the expression of mesenchymal markers, such as N-cadherin, α-SMA and vimentin and decreased the expression of the epithelial marker E-cadherin in MHCC97H cells (Fig. 4a, b). The expression levels of EMT-associated transcriptional regulators (ZEB1, ZEB2 and Slug) were elevated, and the expression level of a MET-associated transcriptional regulator (OVOL1) was inhibited in siRab27a/MHCC97H cells (Fig. 4c). Next, we confirmed the major role of the MAPK/ERK signalling pathway in EMT. Western blot assays showed that depleted expression of Rab27a in MHCC97H cells strongly promoted phosphorylation of Erk1/2, while total levels of Erk1/2 remained unchanged. However, there was no significant change in the expression levels of phospho-Akt and Smad2/3 (Fig. 4d). Treatment with PD98059 markedly abrogated the enhanced phosphorylation of Erk1/2 in siRab27a/MHCC97H cells. Furthermore, the epithelial marker E-cadherin was upregulated, whereas the mesenchymal marker vimentin was downregulated in PD98059-treated siRab27a/MHCC97H cells (Fig. 4e).

Collectively, our findings demonstrate that reduced exosome secretion by Rab27a blockade can promote EMT in parental HCC cells through the MAPK/ERK pathway.

### Expression pattern of Rab27a in human HCCs and its correlation with clinicopathological features

**Fig. 5:**
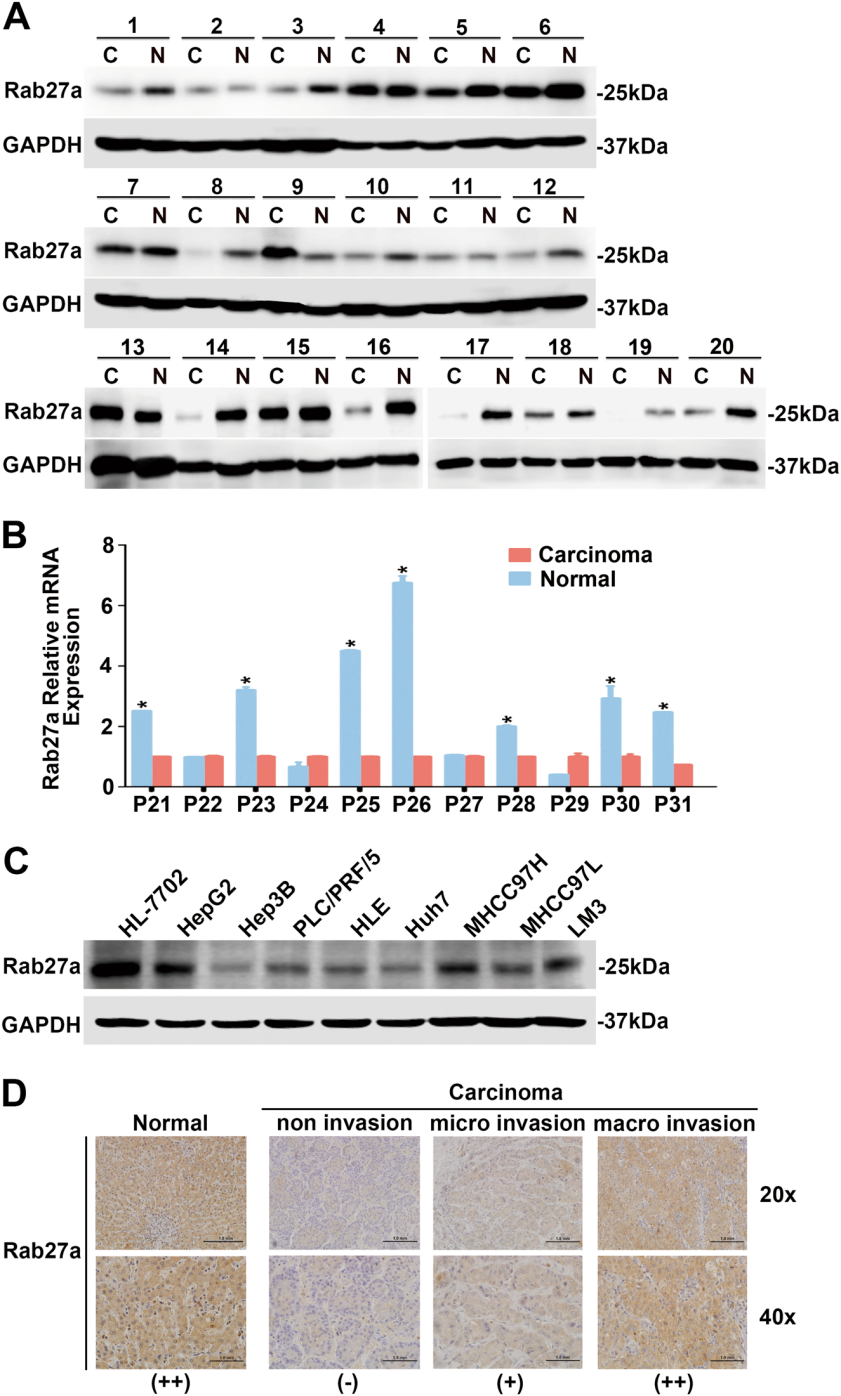
Expression pattern of Rab27a in human HCCs and its correlation with clinicopathological features. **a** Western blot analysis of Rab27a expression in 20 pairs of HCC tissues (C) and non-tumour tissues (N). **b** qRT-PCR of Rab27a expression in 11 pairs of HCC tissues and adjacent non-tumour tissues. **c** Western blot of the protein levels of Rab27a in 7 HCC cell lines and the human HL-7702 hepatocyte cell line. **d** Immunohistochemical staining analysis of Rab27a in non-tumour tissues and HCC tissues with varying degrees of vascular invasion. Three expression levels of staining: negative (−), weakly positive (+) and strongly positive (++); magnifications = ×20, ×40. Scale bar, 1.0 mm

Our research demonstrated the impact of Rab27a on exosome secretion by HCC cells and the functional consequences. Abnormal expression of Rab27a was found in colorectal and pancreatic cancers and was correlated with clinicopathological characteristics and clinical outcomes^27,28^. Therefore, we investigated the expression pattern of Rab27a in HCC samples and its relevance to clinical features of HCC patients. We first examined Rab27a expression in 20 cases of HCC and matched non-tumour tissues by Western blot and another 11 pairs by real-time quantitative RT-PCR (qRT-PCR). The results revealed that Rab27a expression in HCC was substantially lower than that in the adjacent normal liver tissue (15/20, 7/11, respectively) (Fig. 5a, b). Similar results were obtained in HCC cell lines. Low Rab27a expression was observed in all HCC cell lines, whereas HL-7702 had relatively high Rab27a expression. Interestingly, we found that among MHCC97H, MHCC97L and LM3 cells, the expression level of Rab27a was positively correlated with their metastatic potential (LM3>MHCC97H>MHCC97L; Fig. 5c)^29^. Immunohistochemical (IHC) analysis of 67 HCC tissues and matched non-tumour tissues indicated that the latter samples showed higher levels of Rab27a than the former ones. In addition, among tumour samples, the staining intensity of Rab27a was increased in HCC with vascular invasion (Fig. 5d). Clinicopathological analysis showed that Rab27a expression was linked with elevated serum AFP (*P* = 0.009), vascular invasion (*P* = 0.031) and liver cirrhosis (*P* = 0.035) (Table. 1). However, for the prognostic value of Rab27a, we found that there was no statistically significant difference in 3-year overall survival rate between positive and negative Rab27a expression groups (*P* > 0.05, Supplementary Figure S4). Thus, our analysis clearly indicates that the high Rab27a expression is associated with tumour progression and metastasis.

**Table 1:** Clinicopathological characteristics of patients with different Rab27a expression levels undergoing surgery for hepatocellular carcinoma

Clinicopathological characteristics	*N* (%)	Rab27a expression^a^		*P* value
		Low expression	High expression	
Tumour size (cm)				
≤5	43	17	26	.283
>5	24	12	12	
Tumour number				
Single	54	26	28	.091
Multiple	13	3	10	
AFP^b^				
(−)	44	14	30	.009*
(+)	23	15	8	
HBV				
(−)	18	7	11	.43
(+)	49	22	27	
HCV				
(−)	61	25	36	.217
(+)	6	4	2	
Satellite nodule				
(−)	47	21	26	.468
(+)	20	8	12	
Vascular invasion				
(−)	63	25	38	.031*
(+)	4	4	0	
Liver cirrhosis				
(−)	35	11	24	.035*
(+)	32	18	14	
Tumour thrombus				
(−)	24	9	15	.325
(+)	43	20	23	

*HBV* hepatitis B virus, *HCV* hepatitis C virus

*P < 0.05 was considered statistically significant

^a^Final staining score ≤ 6 was defined as low expression; final staining score > 6 was defined as high expression

^b^AFP (−) ≤500, AFP (+) >500

### Inhibition of self-derived exosome secretion or exogenous exosome uptake enhances HCC metastasis and recurrence in vivo

**Fig. 6:**
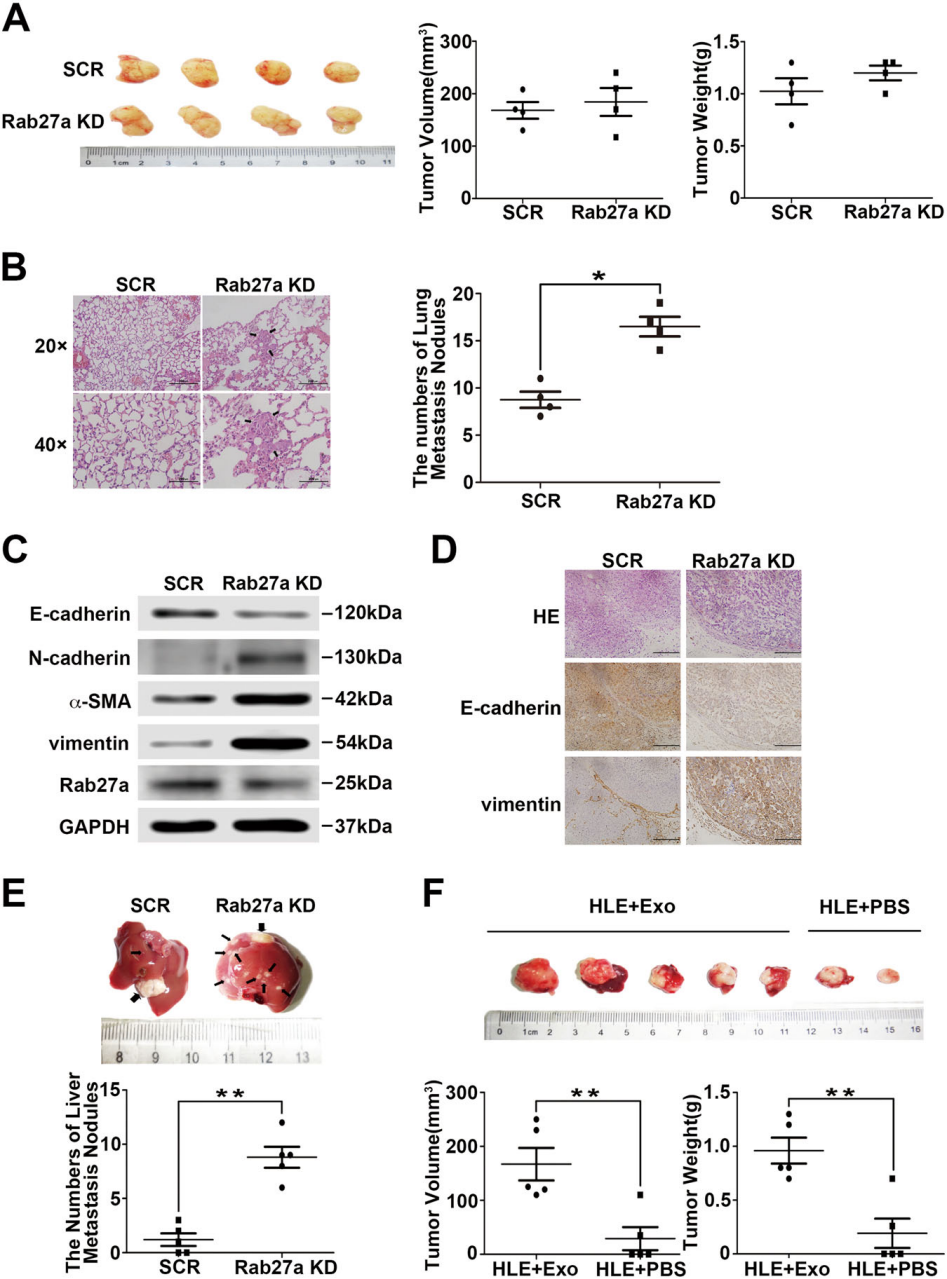
Inhibition of self-derived exosome secretion or exogenous exosome uptake enhances HCC metastasis and recurrence in vivo. **a** Comparison of tumour size and weight in nude mice subcutaneously implanted with siSCR/MHCC97H or siRab27a/MHCC97H cells (*n* = 4/group). **b** The lung metastatic ability of human tumours in the siSCR/MHCC97H cell-injection and siRab27a/MHCC97H cell-injection groups visualised using H&E staining, magnifications = ×20, ×40. The arrow indicates the metastatic foci. **c** Western blot analysis of Rab27a and EMT markers in subcutaneously formed tumours in the siSCR/MHCC97H cell-injection and siRab27a/MHCC97H cell-injection groups. **d** Immunohistochemical assay of E-cadherin and N-cadherin in subcutaneously formed tumours in the siSCR/MHCC97H cell-injection and siRab27a/MHCC97H cell-injection groups. **e** The intrahepatic metastatic ability of siRab27a/MHCC97H and siSCR/MHCC97H cells (*n* = 5). The thick arrow indicates the orthotopically implanted tumours, and the thin arrow indicated the intrahepatic metastatic foci. **f** The effect of MHCC97H-derived exosomes on intrahepatic recurrence of HLE tumours (*n* = 5). Abbreviation: Exo exosome. Data are represented as the mean ± S.D. *P < 0.05 and **P < 0.01. Scale bar, 1.0 mm

To evaluate the potential contribution of exosomes to the metastasis and recurrence of HCC in vivo, we first investigated the impact of reduced self-derived exosome secretion caused by Rab27a knockdown on HCC metastasis using a mouse model. siRab27a/MHCC97H and siSCR/MHCC97H cells were subcutaneously injected into the groins of nude mice (5 × 10^6^/mouse). Six weeks after inoculation, when the first mouse was about to die, all mice in the two groups were sacrificed, and tumour sizes and weights were evaluated. No significant differences in tumour sizes and weights were observed between the two groups (Fig. 6a), which confirmed the result of the CCK8 proliferation assays in vitro (Supplementary Figure S2). However, Rab27a knockdown substantially increased the number of pulmonary metastatic foci (*P* < 0.05; Fig. 6b). Consistent with the previous in vitro experimental results, the expression of the epithelial marker E-cadherin was decreased in siRab27a/MHCC97H tumour tissues, accompanied by increased levels of the mesenchymal markers N-cadherin, α-SMA and vimentin (Fig. 6c). These results were verified by IHC assays (Fig. 6d). To better imitate the microenvironment of HCC cells in the liver and determine the effect of decreased exosome secretion by Rab27a downregulation on intrahepatic metastasis of HCC cells, we established orthotopic xenografts of HCC tumours. The two types of subcutaneously formed tumours were cut into small pieces (2 × 2 mm) and were anchored to the liver surface of two groups of new nude mice. Three weeks later, we resected the livers and found that in the siRab27a/MHCC97H tumour group, all the mice (5/5, 100%) developed micrometastatic lesions throughout the liver, while only three mice (3/5, 60%) had a few micrometastatic lesions in the control group (*P* < 0.01; Fig. 6e).

Then, we explored the effect of tumour-derived exosomes on HCC recurrence in vivo. Similarly, we established orthotopic xenografts of HLE tumours (*n* = 5/group, two groups). Four weeks later, the orthotopically engrafted tumours were resected. The surgical margin was 2 mm larger than the tumour edge to ensure complete resection. One week later, the experimental group was intravenously (tail vein) injected with MHCC97H-derived exosomes (100 μg/mouse), while the control group was injected with the same volume of PBS. The injection was repeated once a week. After 4 weeks, all mice were sacrificed and the livers were resected. Notably, 5/5 (100%) mice in the exosome injection group showed intrahepatic recurrence in the remaining liver, while the recurrence rate was only 40% (2/5) in the PBS injection group. Moreover, the tumour size and weight were greater in the exosome injection group than the control group (*P* < 0.01; Fig. 6f).

Thus, we conclude that inhibition of self-derived exosome secretion by Rab27a blockade promotes intrahepatic and lung metastasis of MHCC97H tumours. MHCC97H-derived exosomes can enhance HLE-originated HCC recurrence after surgical resection.

### Ectopic overexpression of Rab27a rescues HCC metastasis in vivo

**Fig. 7:**
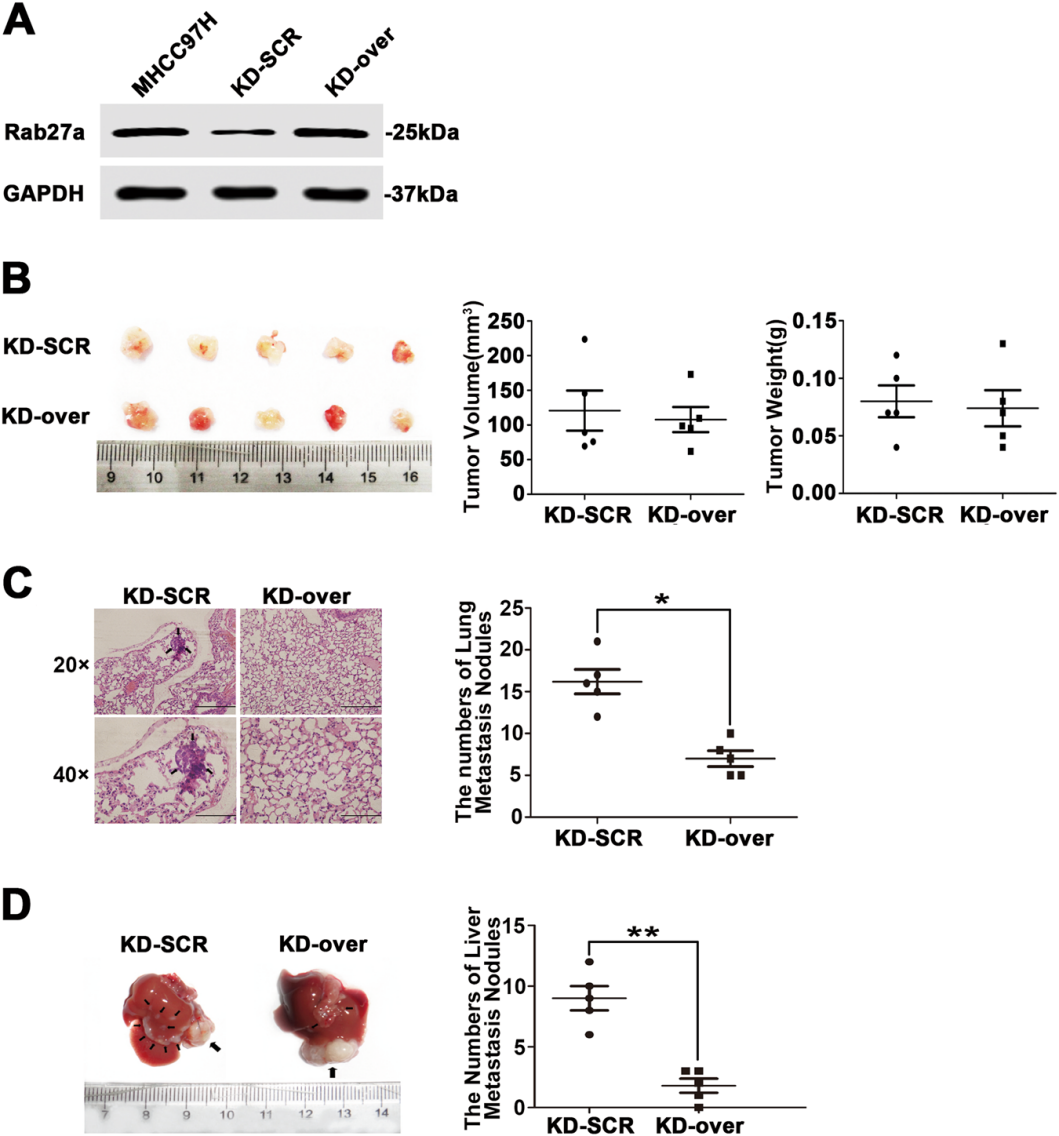
Ectopic overexpression of Rab27a rescues HCC metastasis in vivo. **a** The efficiency of Rab27a overexpression was examined by Western blot. **b** Comparison of tumour size and weight in nude mice subcutaneously implanted with overSCR-siRab27a/MHCC97H or overRab27a-siRab27a/MHCC97H cells (*n* = 5/group). **c** The lung metastatic ability of human tumours in the overSCR-siRab27a/MHCC97H cell-injection and overRab27a-siRab27a/MHCC97H cell-injection groups visualised using H&E staining, magnifications = ×20, ×40. The arrow indicates the metastatic foci. **d** The intrahepatic metastatic ability of overSCR-siRab27a/MHCC97H and overRab27a-siRab27a/MHCC97H cells (*n* = 5). The thick arrow indicates the orthotopically implanted tumours and the thin arrow indicated the intrahepatic metastatic foci. Abbreviation: KD-SCR overSCR-siRab27a/MHCC97H; KD-over overRab27a-siRab27a/MHCC97H. Data are represented as the mean ± S.D. *P < 0.05 and **P < 0.01. Scale bar, 1.0 mm

To better understand the role of MHCC97H-derived exosomes in the lung and intrahepatic metastasis, we performed another group of animal experiment. Firstly, we stably overexpressed Rab27a in siRab27a/MHCC97H cells using specific plasmid lentiviral infection. The efficiency was confirmed by Western blot (Fig. 7a). Then, 5 × 10^6^ cells/0.2 ml of overSCR-siRab27a/MHCC97H or overRab27a-siRab27a/MHCC97H cells were subcutaneously transplanted into the right groins of four-week-old nude mice (*n* = 5/group). Both groups of mice were killed 3 weeks later. We checked tumour volumes and weights, as well as the number of lung metastasis nodules. The result showed that no significant differences in tumour sizes and weights were observed between the two groups (Fig. 7b). However, Rab27a overexpression significantly rescued the lung metastases displayed by xenografted mice with siRab27a/MHCC97H cells. (*P* < 0.05; Fig. 7c). Furthermore, orthotopic tumour model of HCC in mice was established using the above subcutaneously formed tumours. Three weeks later, the mice were sacrificed and the livers were resected. We found that upregulation of Rab27a repressed the increase of intrahepatic metastases in siRab27a/MHCC97H-related xenografted mice (Figs. 6e and 7d). This phenomenon further verified that Rab27a-dependent exosome secretion was vital for HCC metastasis.

Taken together, we believe that ectopic overexpression of Rab27a in MHCC97H cells can rescue the lung and intrahepatic metastases displayed by xenografted mice with siRab27a/MHCC97H cells.

## Discussion

Exosomes carry a functionally active cargo of proteins, RNAs and other types of molecules and have been implicated in the pathology of cancer^30^. Exosomes secreted from breast cancer cells contain miRNAs involved in cancer progression and can induce non-tumorigenic mammary epithelial cells to form tumours^5^. Macrophage migration inhibitory factor in pancreatic cancer-derived exosomes can prime the liver for metastasis^31^. Recently, the role of exosomes in HCC development and progression has been widely investigated^32^. In this study, we demonstrated that exosomes derived from highly metastatic MHCC97H cells could be taken up by low metastatic HCC cells and subsequently promoted malignant behaviours of the recipient cells. These results are consistent with a previous study that revealed that malignant tumour cells can enhance the migratory and metastatic capacities of less malignant cells through transfer of extracellular vesicles in living mice^18^. Tumour invasion and metastasis are major causes of early recurrence after hepatectomy^3^. Previous data have shown that the most frequent site of recurrence is the remnant liver, which accounts for approximately 85–90% of the initial recurrences^33^. Notably, we found that injection of MHCC97H-derived exosomes through the tail vein significantly facilitated tumour recurrence in the remnant liver after surgical resection of orthotopically formed HLE tumours. These findings suggest that elimination of exosomes in the circulation might be a promising strategy along with hepatectomy to improve the survival rate of HCC patients.

Based on the enhancement of malignancy in HLE and Hep3B cells by treatment with MHCC97H-derived exosomes, we hypothesised that exosomes released from highly metastatic HCC cell lines may contain biological molecules important for cancer invasion and metastasis. This hypothesis is consistent with a previous report showing that exosomes derived from highly metastatic MHCC97L and HKCI-8 cells carry many pro-tumorigenic RNAs and proteins^34^. We then investigated whether the pro-malignant factors transferred to recipient cells by exosome treatment can be reserved in donor cells once exosome exocytosis is blocked. Rab27a is required for efficient secretion of exosomes^35^. We showed for the first time that MHCC97H cells secreted exosomes in a Rab27a-dependent manner. As expected, functional analysis revealed that migration, chemotaxis and invasion were elevated in MHCC97H cells after reducing exosome secretion by Rab27a knockdown. Orthotopically implanted siRab27a/MHCC97H cells could form more intrahepatic and lung metastatic foci than control cells in vivo. In contrast, some previous studies showed that reduced exocytosis of miRNAs by Rab27a knockdown caused intracellular upregulation of active tumour-suppressor miRNAs and consequently inhibited tumour growth and invasion^12,36^. This discrepancy may be attributed to the various contents of exosomes, which require further exploration. Moreover, among the LM3, MHCC97H and MHCC97L cell lines, the expression levels of Rab27a were positively correlated with the metastatic potential of HCC cell lines (Fig. 5c). We concluded that this phenomenon might explain the increased secretion of exosomes with metastatic properties by HCC cells with higher level of Rab27a. The positive correlation between Rab27a and serum AFP, as well as vascular invasion may further support the idea that elevated exosome secretion indicates a more aggressive form of HCC.

EMT is involved in pathological processes of HCC, including anti-apoptosis effects, invasion, metastasis and chemoresistance^37,38^. Recent studies revealed that tumour-derived exosomes contain pro-EMT factors and thereby facilitate tumour progression^39^. However, there are few studies examining how HCC-derived exosomes induce plasticity through EMT in the tumour microenvironment. Our data showed that MHCC97H-secreted exosomes could induce recipient HLE and Hep3B cells to undergo EMT. Blocking exosome release by Rab27a knockdown can also promote EMT in parental MHCC97H cells. Furthermore, we also demonstrated that the MAPK/ERK pathway was involved in EMT induced by incubation of HLE cells with MHCC97H-derived exosomes and reduction of exosome secretion in parental MHCC97H cells by Rab27a knockdown. These results support the finding that HCC-derived exosomes can promote EMT through the MAPK signalling pathway. Exosomes released by gastric cancer cells enhanced tumour proliferation by activating the MAPK/ERK pathway^40^. Increased ERK activity was also observed in immortalised hepatocyte cocultured with HCC-derived exosomes^34^. This may be one of the mechanisms underlying tumour exosome-mediated cancer progression, and therefore, it may be applicable for the treatment of HCC.

In conclusion, our work identifies a possible role of tumour-derived exosomes in promoting the EMT process through MAPK/ERK signalling, which further facilitates HCC invasion and metastasis. We also uncover a novel potential mechanism for intrahepatic recurrence. This study does not discuss the specific responsible exosomal molecules; therefore, additional research is needed to address this issue. These findings may contribute to the identification of new therapeutic targets and prognostic prediction markers, as well as the development of treatment strategies.

## Materials and methods

### Patients and specimens

Twenty-three cases of tumours and adjacent non-cancerous liver tissues and 67 cases of paraffin-embedded tissue samples were collected from HCC patients, who underwent surgical resection at Tianjin Medical University Cancer Hospital (Tianjin, China) from January 2013 to January 2014. Informed consent was obtained from all the patients involved. This study was conducted in accordance with Declaration of Helsinki and was approved by the Tianjin Medical University Cancer Hospital Ethics Committee. All tumour samples were histologically assessed for diagnosis, tumour samples were confirmed with more than 70% viable tumour cells and non-cancerous liver samples were confirmed to be free of tumour cells.

### Cell lines and cell culture

The human HL-7702 hepatocyte cell line and human HCC cell lines, MHCC97H, MHCC97L and LM3 were obtained from the Liver Cancer Institute, Zhongshan Hospital, Fudan University, Shanghai, China. Some other human HCC cell lines, HepG2, Hep3B, PLC/PRF/5 and HEK-293T cells were purchased from American Type Culture Collection (ATCC; Manassas, VA, USA). Huh7 cells were bought from the Health Science Research Resources Bank (Shanghai, China). HLE cells from the Health Science Research Resources Bank (Osaka, Japan) were also used in this study. HL-7702, MHCC97H, MHCC97L, LM3, Huh7, HLE and 293T cells were cultured in Dulbecco’s Modified Eagle’s Medium (DMEM) (Gibco, Carlsbad, CA, USA), and HepG2, Hep3B and PLC/PRF/5 cells in Eagle’s Minimum Essential Medium (Gibco), containing 10% foetal bovine serum (FBS; HyClone Laboratories Inc., Novato, CA, USA) and 1% penicillin-streptomycin solution (PS; Hyclone), at 37 °C with 5% CO_2_. PD98059 (25 μM, Sigma-Aldrich, St. Louis, MO, USA) was an inhibitor of ERK.

### Western blot assay

The cells were lysed in ice-cold lysis buffer, then the lysed proteins were separated by SDS-PAGE gel, followed by being transferred onto a polyvinylidene difluoride membrane (Immobilon-P, Millipore, Billerica, MA, USA). Antibodies against the following proteins were used: TSG101(51: sc-136111), GRP78(A-10: sc-376768), GAPDH (0411: sc-47724), N-cadherin (H-63: sc-7939), CD63 (H-193: sc-15363), ZEB1 (E-20: sc-10572), ZEB2 (E-11: sc-271984), Slug (A-7: sc-166476) (Santa Cruz Biotechnology, CA, USA), vimentin (2707-1) (Epitomics, Burlingame, CA, USA), E-cadherin (610181), Smad2/3 (610842) (BD Biosciences, San Jose, CA, USA), Alix (2171), p-AKT (Ser473) (4060), AKT (9272), p-Smad2 (Ser465/467) /Smad3 (Ser423/425) (8828), p-Erk1/2 (Thr202/Tyr204) (4370), Erk1/2 (9102) (Cell Signalling Technologies, Danvers, MA, USA), Rab27a (ab55667), α-SMA (ab5694), Glypican 3 (ab66596) (Abcam, Hong Kong, China), AFP (14550-1-AP), OVOL1 (14082-1-AP) (Proteintech, Chicago, IL, USA) overnight at 4 °C. Afterwards, HRP-conjugated anti-mouse (Santa Cruz, sc-2005) or anti-rabbit (Santa Cruz, sc-2004) secondary antibodies were used to incubate with the membrane. Blots were visualised using enhanced chemiluminescence reagents ECL (Pierce, Rockford, IL).

### Cell transfection

Transfection was performed with the Lenti-PacTM HIV Expression Packaging Kit (GeneCopoeia, Rockville, MD, USA). The procedures of transfection were followed according to the manufacturer’s instruction. MHCC97H cells were transduced with the HIV vector at MOI 10. A Rab27a-specific shRNA (sequence: GATCAGTTAAGTGAAGAAA) and a scrambled sequence of shRNA to GFP as negative control (SCR) were bought from Genechem (Shanghai, China). Stable cell lines were selected using puromycin (Gibco)-containing medium. The efficiency of stably transfected cell clone was confirmed by real-time qRT-PCR and western blot.

### IHC assay

Paraffin-embedded tissues of HCC, which had been confirmed by Hematoxylin and Eosin (H&E) staining, were obtained from Tianjin Medical University Cancer Institute and Hospital. Anti-Rab27a antibody (Abcam) was incubated with HCC tissues for 72 h at 4 °C. The incubation with the E-cadherin (BD Biosciences) or vimentin (Epitomics) antibodies occurred at room temperature for half an hour and then at 4 °C overnight. Then staining with the secondary antibody PV-6002 Kit (Zhongshan Golden Bridge Biotechnology, Beijing, China) for 1 h at 37 °C was performed. Finally, reaction products were visualised with 3, 3′-diaminobenzidine tetrahydrochloride and counterstained with 10% Mayer hematoxylin.

The immunostaining intensity of Rab27a was scored as: 0, negative; 1, weak; 2, moderate; 3, strong. The percentage of Rab27a-positive cells was scored on a scale from 0 to 3 (0 for no positive cells, 1 for <30% positive cells, 2 for 30–60% positive cells and 3 for >60% positive cells). The sum of the intensity and percentage scores was used as the final staining score. We finally classified three expression levels of the staining: negative (−) (score = 0–1); weakly positive (+) (score = 2–6); strong positive (++) (score = 7–9). All images were captured with political fluorescence microscope (Olympus BX61, Tokyo, Japan).

### RNA isolation and qRT-PCR

Total RNA was extracted using Trizol (Invitrogen, Carlsbad, CA, USA). The extracted RNA was quantified using NanoDrop ND-1000 (NanoDrop Technologies, Wilmington, DE, USA). Then, complementary single strand DNA (cDNA) was synthesised using PrimeScript^TM^ RT Master Mix (TaKaRa Biotechnology, Dalian, China). For the quantitative PCR, SYBR^®^ Premix Ex Taq^TM^ II (Tli RNaseH Plus) was used (TaKaRa Biotechnology). The PCR primers were as follows: Rab27a (forward: 5′-GATGCTTCTGGACCTGATAATGA-3′, reverse: 5′-TGCCCCTTTCTCCTTTTCTTC-3′) and GAPDH (forward: 5′-TGGTATCGTGGAAGGACTCA-3′, reverse: 5′-CCAGTAGAGGCAGGGATGAT-3′). Three independent experiments were performed, Data are shown as mean ± SD.

### Proliferation assay

Cells were plated in 96-well plates at a density of 2 × 10^3^ cells per well. To reduce differences within the group, each group of cells sample a set of five parallel holes. Then, the cells were incubated with Cell Counting Kit-8 (Dojindo Laboratories, Kumamoto, Japan; 10 μl/well) for 4 h in 37 °C and 5% CO_2_. The optical density was measured by an ELISA reader (BioTek Synergy H1, Burlington, VT, USA).

### Scratch assay

1 × 10^6^ cells/well were plated in a six-well plate. The other day, the cells formed a monolayer. A 10 μl pipette tip was used to make a straight scratch. The culture medium was changed to DMEM with 0.5% FBS. The cells were incubated in a 37 °C humidified incubator with 5% CO_2_. The wound distance was measured in a light microscope every 6 h, the total time was 24 h. All samples were tested in triplicate and the data are expressed as the mean ± SD.

### Chemotaxis assay

Briefly, 30 μl DMEM medium containing 20% FBS was loaded into the lower chamber. Fifty microlitres cells (5 × 10^5^/ml) suspended in DMEM-only medium were loaded into the upper chamber. A 8 μm polycarbonate filter was pretreated with 0.001% fibronectin, which was diluted by 0.1% fibronectin (Sigma-Aldrich) in DMEM at 4 °C overnight. Then the membrane was put between the two chambers. After incubating the whole device in 5% CO_2_ at 37 °C for 24 h, the membrane was washed, fixed and stained. The number of migrated cells were counted in three randomly chosen high-power fields (×400) by light microscopy. All samples were tested in triplicate and the data are expressed as the mean ± SD.

### Matrigel invasion assay

Briefly, the transwell chambers (Costar, Cambridge, MA) with 8 μm pore size were coated with diluted Matrigel (1:3 by DMEM-only medium, BD). Then 200 μl of cells (1 × 10^6^/ml) suspended in DMEM-only medium were loaded in triplicate upper chambers. Six hundred microlitres DMEM medium with 20% FBS was added into the lower chamber. After incubated 36 h in a 37 °C humidified incubator with 5% CO_2_, the invaded cells were fixed, washed and stained. Three high-power fields (×400) were randomly chosen to count the cell number. All samples were tested in triplicate and the data are expressed as the mean ± SD.

### Colony formation assay

Cells were seeded into 6-well plates (500 cells per well) and cultured for 2 weeks. The colonies on the plate were fixed with 4% paraformaldehyde (PFA) and stained with 0.02% crystal violet. The numbers of colonies were counted.

### 3D sphere formation assay

The 3D cell culture chambers (Thermo fisher Scientific, Waltham, MA, USA) were pre-coated with Matrigel (100 μl per well, BD). Four hundred microlitres cells suspended in 0.2% Matrigel (diluted by DMEM only) was loaded in the chambers (5000 cells per well). The supernatant medium was changed every three days. After incubated for 3 weeks, the numbers of colonies were counted in three randomly chosen fields (×10) using inverted microscope (Leica DMI6000B).

### Immunofluorescence and confocal microscopy

Pretreated sterile coverslips were lay to the 12-well plate, then 5 × 10^4^ cells were plated in every well, followed by being incubated for 12 h at 37 °C in 5% CO_2_. Afterwards, the cells were fixed and permeabilized before being incubated with primary antibodies overnight at 4 °C. The types and concentrations of primary antibodies are as followed: E-cadherin (1:20) (BD Biosciences), α-SMA (1:100) (Abcam) and vimentin (1:5000) (Epitomics). After being washed twice with PBS, the cells were stained with an Alexa Fluor 594-conjugated (Life Technologies, Carlsbad, CA, USA) secondary antibody at room temperature for 1 h in a dark box. Cell nuclei were counterstained with prolong gold anti-fade reagent (Invitrogen). Fluorescence of cells was visualised with a confocal laser scanning microscope (Olympus FV1000).

### Isolation of exosomes

In this study, differential centrifugation was used to purify exosomes from cell culture supernatant. Briefly, the cell culture medium was collected and centrifuged at 1000×*g* for 15 min to remove whole cells. The supernatant was then centrifuged at 2500×*g* for 15 min to remove cell debris, which was followed by centrifugation at 10,000×*g* for 30 min to remove debris and large vesicles. The supernatant was collected and filtered with a 0.22-μm filter (Millex, Germany), followed by ultracentrifugation at 100,000×*g* for 70 min to pellet exosomes. Exosome pellets were washed in a large volume of PBS and recovered by centrifugation at 100,000 g for 1 h. The total protein concentration of exosomes was quantified by the Bradford assay (Sangon Biotech, Shanghai, China). The size distribution of the exosome sample was measured by Zetasizer Nano ZS90 (Malvern, Worcestershire, UK)

### Transmission electron microscopy

Briefly, a 10 μl drop of exosomes pre-fixed with 4% PFA was loaded onto a formvar-carbon-coated grid and incubated for 20 min. After washed by PBS, the grid was refixed by 1% glutaraldehyde in 0.1 M sodium phosphate buffer (pH 7.4) for 5 min. The grid was washed for 8 times with distilled water. The grid was contrasted first in a solution of uranyl oxalate, pH 7 and then contrasted and embedded in a mixture of 4% uranyl acetate and 2% methyl cellulose in a ratio of 100 μl/900 μl, respectively. The grid was dried in the air for 5–10 min. The grid was observed under the electron microscope at 80 kV.

### Labelling of exosomes

Purified exosomes were labelled using Exo-Glow™ Exosome Labelling Kit (System Biosciences, Mountain View, CA, USA). Briefly, 50 μl EXO-Red (labelling single-stranded RNAs) or 50 μl EXO-Green (labelling proteins) was added to 500 μl of purified exosomes. After incubated at 37 °C for 10 min, the labelling reaction was stopped by ExoQuick-TC reagent. The labelling exosome sample was placed on ice for 30 min, followed by centrifugation for 3 min at 14,000 rpm. Hundred mictolitres of labelled exosomes were added to 1 × 10^5^ cells per well in a 6-well plate. After incubated for 24 h, the cells were imaged using a confocal microscope to confirm the presence of exosomes within the cells.

### Animal experiments

The mice were purchased from Nanjing model animal research center. All animal work procedures were approved by the Ethics Committee of the Tianjin Medical University Cancer Institute and Hospital, China. siSCR/MHCC97H or siRab27a/MHCC97H cells were subcutaneously inoculated into the right groins of the nude mice (male, 4 weeks old, *n* = 4/group) at a density of 5 × 10^6^ in 0.2 ml of PBS. At the sixth week after injection, both groups of mice were killed when the first mouse became moribund. Tumours were measured by using a caliper and were weighted. The tumour volume was calculated by: (large diameter) × (small diameter)^2^/2. In order to examine the numbers of lung metastasis nodules, the lungs were embedded in paraffin and serially resected for H&E staining. Some of tumour tissues were frozen at −80 °C for western blot analysis. Then orthotopically implanted tumours were formed. Briefly, two tumours from the two groups were randomly chosen and cut into small pieces (2 × 2 mm) and subsequently anchored to the liver parenchyma of new nude mice (*n* = 5/group) under anaesthesia. Three weeks later, the mice were sacrificed and the livers were resected.

In order to figure out the role of exosomes in HCC recurrence in vivo, HLE-derived subcutaneously formed tumour was cut into pieces (2 × 2 mm) and anchored to the liver of two groups of new nude mice (*n* = 5/group) under anaesthesia. Four week later, the tumour in the liver was resected. The surgical margin should be free of tumour cells. One week later, 100 μg of MHCC97H-derived exosomes were intravenously injected to the mice (tail vein), while the control group was injected with the same volume of PBS. The injection was repeated one week later. Four weeks later, the mice were sacrificed and the liver was resected.

### Statistical analysis

All statistical analyses were performed using the statistical software SPSS 22.0 (SPSS Inc., Chicago, IL). Overall survival rate was calculated by the Kaplan–Meier method and the log-rank test was used for single-factor analysis. Overall survival was calculated from the date of surgery to the time of death from any cause or until the last follow-up. Two-tailed *P* *<* 0.05 was considered statistically significant.

## Electronic supplementary material

Supplementary Figure 1

Supplementary Figure 2

Supplementary Figure 3

Supplementary Figure 4

Supplementary Material
