# Supplementary material for: HCC-derived exosomes elicit HCC progression and recurrence by epithelial-mesenchymal transition through MAPK/ERK signalling pathway
Source: Cell Death Dis. 2018 May 3;9(5):513. doi: 10.1038/s41419-018-0534-9 (PMC5938707; doi:10.1038/s41419-018-0534-9)

**Figure S2 MHCC97H-derived exosomes promote migration and invasion as well as EMT process in Hep3B cells.** (A) Scratch assay for the migration of Hep3B cells treated with or without MHCC97H-derived exosomes (100 μg/ml). The distance was measured every 3 h for 24 h. (B) The chemotactic potential of Hep3B cells treated with or without MHCC97H-derived exosomes (100 μg/ml). The incubation time was 24 h. (C) Matrigel invasion assay for the invasion of Hep3B cells treated with or without MHCC97H-derived exosomes (100 μg/ml). The incubation time was 36 h. (D) Colony formation assay of Hep3B cells treated with or without MHCC97H-derived exosomes (100 μg/ml). The culture time was 2 weeks. (E) Western blot analysis of EMT markers in Hep3B cells treated with or without MHCC97H-derived exosomes for 24 h. Abbreviation: Exo, exosome. **P* < 0.05, ***P* < 0.01 and ****P* < 0.001. Scale bar, 1.0 mm. Data are represented as the mean ± S.D. All experiments were repeated at least three times.


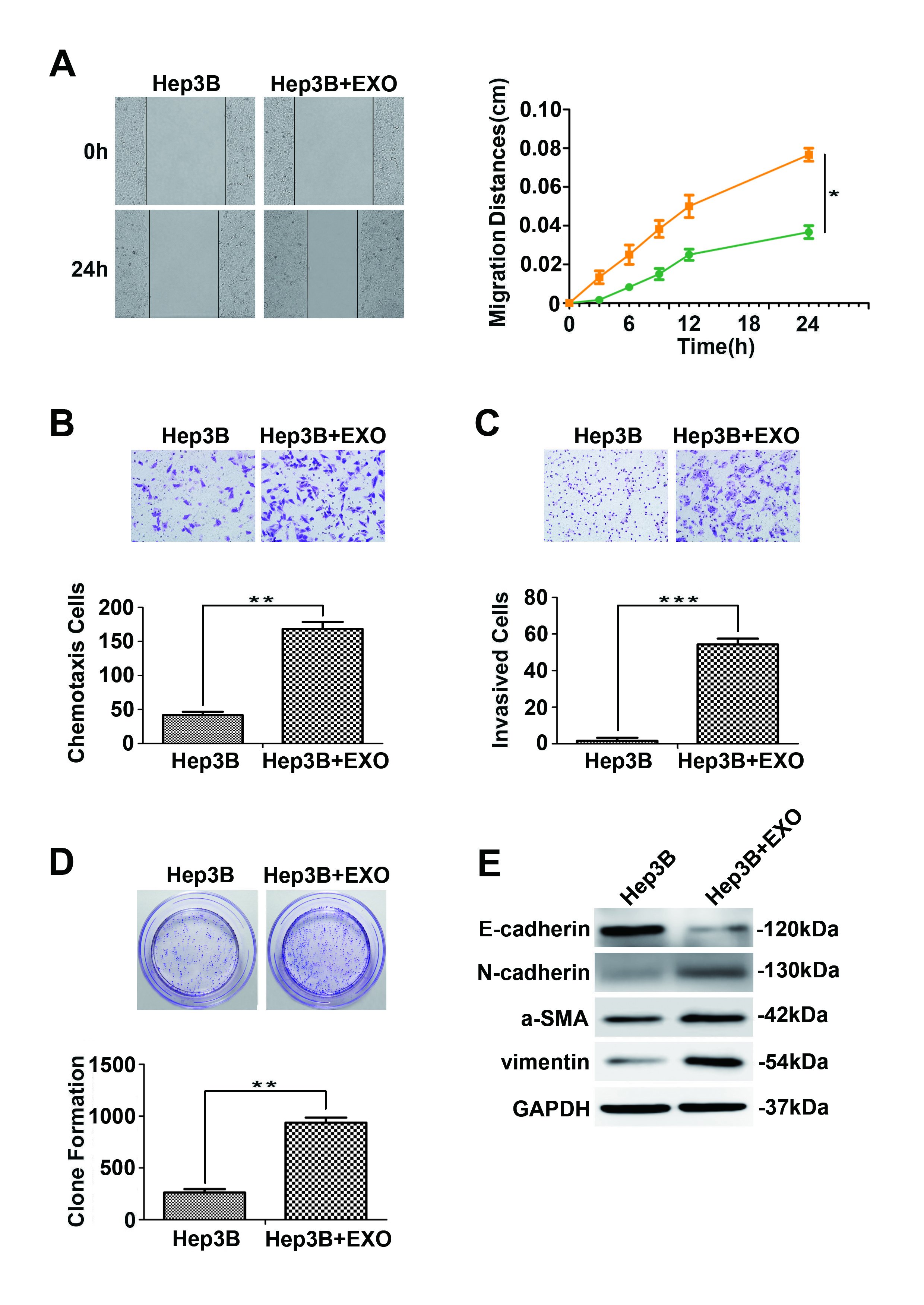

Supplement: Supplementary file 2 — Supplementary Figure 2 [file 41419_2018_534_MOESM2_ESM.docx]
