# Supplementary material for: HCC-derived exosomes elicit HCC progression and recurrence by epithelial-mesenchymal transition through MAPK/ERK signalling pathway
Source: Cell Death Dis. 2018 May 3;9(5):513. doi: 10.1038/s41419-018-0534-9 (PMC5938707; doi:10.1038/s41419-018-0534-9)

**Figure S3** Comparison of the proliferation ability of MHCC97H (WT), siSCR/MHCC97H and siRab27a/MHCC97H cells by Cell Counting Kit-8 (CCK8) assay.
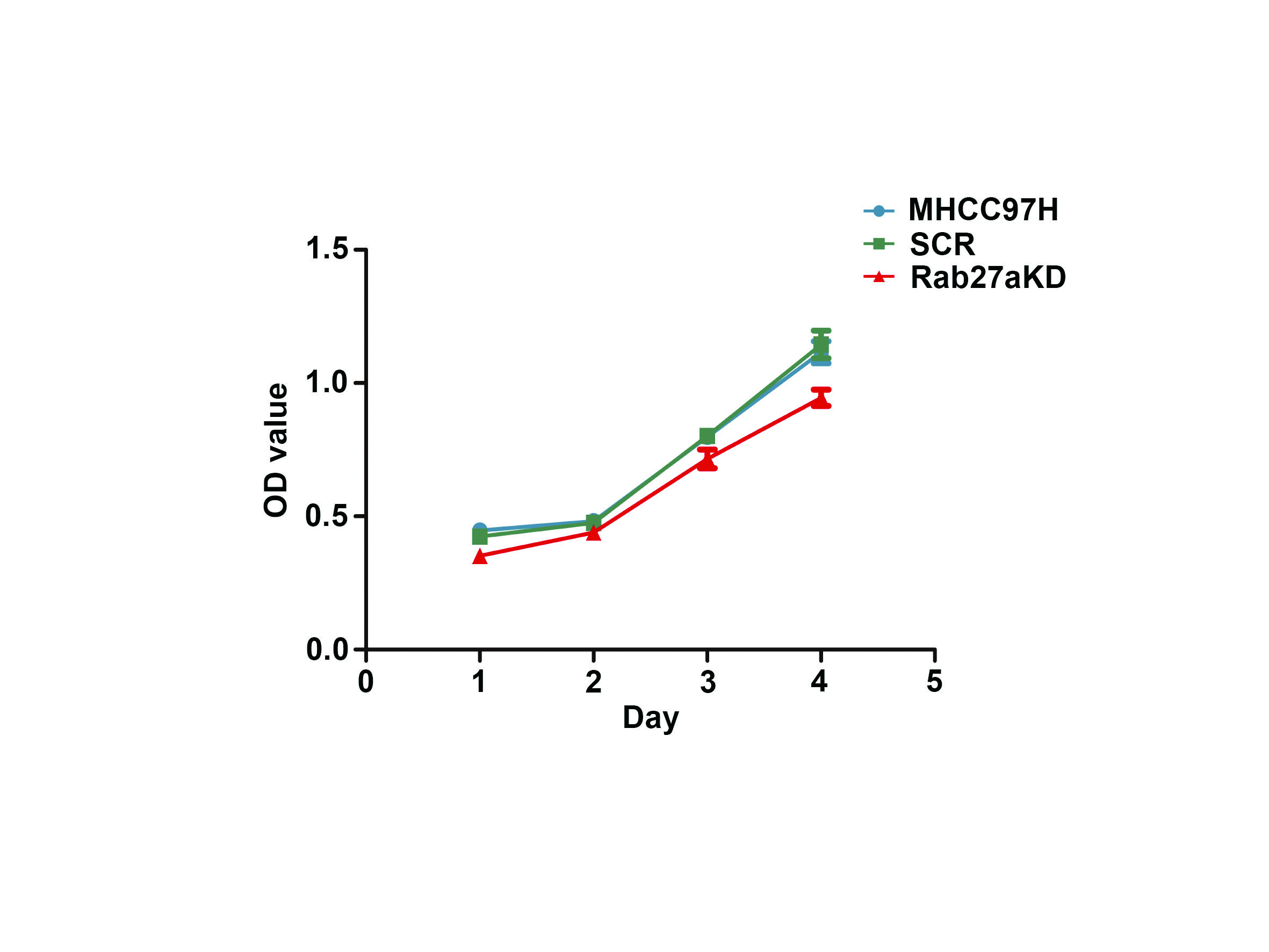

Supplement: Supplementary file 3 — Supplementary Figure 3 [file 41419_2018_534_MOESM3_ESM.docx]
