# Supplementary material for: HCC-derived exosomes elicit HCC progression and recurrence by epithelial-mesenchymal transition through MAPK/ERK signalling pathway
Source: Cell Death Dis. 2018 May 3;9(5):513. doi: 10.1038/s41419-018-0534-9 (PMC5938707; doi:10.1038/s41419-018-0534-9)

**Figure S4** Kaplan-Meier overall survival analysis of 67 HCC patients with negative (-) or positive (+) Rab27a expression.


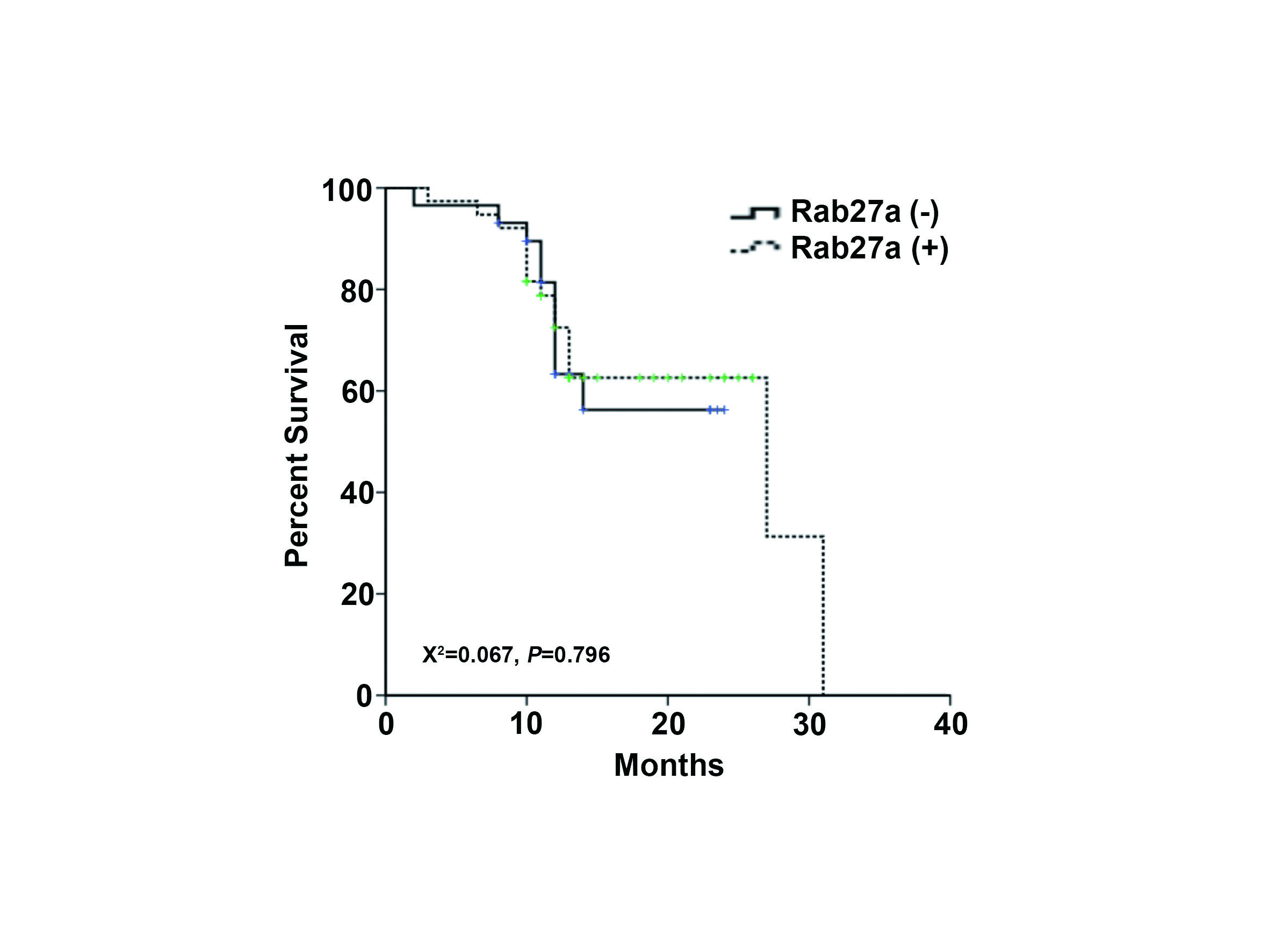

Supplement: Supplementary file 4 — Supplementary Figure 4 [file 41419_2018_534_MOESM4_ESM.docx]
