# Supplementary material for: HCC-derived exosomes elicit HCC progression and recurrence by epithelial-mesenchymal transition through MAPK/ERK signalling pathway
Source: Cell Death Dis. 2018 May 3;9(5):513. doi: 10.1038/s41419-018-0534-9 (PMC5938707; doi:10.1038/s41419-018-0534-9)
Supplement: Supplementary file 5 — Supplementary Material [file 41419_2018_534_MOESM5_ESM.pdf]

Nature Research Editing Service Certification

This is to certify that the manuscript titled Manuscript “HCC-derived exosomes drive HCC progression and recurrence by epithelial-mesenchymal transition through the MAPK/ERK signalling pathway” was edited for English language usage, grammar, spelling and punctuation by one or more native English-speaking editors at Nature Research Editing Service. The editors focused on correcting improper language and rephrasing awkward sentences, using their scientific training to point out passages that were confusing or vague. Every effort has been made to ensure that neither the research content nor the authors' intentions were altered in any way during the editing process.

Documents receiving this certification should be English-ready for publication; however, please note that the author has the ability to accept or reject our suggestions and changes. To verify the final edited version, please visit our verification page. If you have any questions or concerns over this edited document, please contact Nature Research Editing Service at support@as.springernature.com.

Manuscript title: Manuscript “HCC-derived exosomes drive HCC progression and recurrence by epithelial-mesenchymal transition through the MAPK/ERK signalling pathway”

Authors: Lu Chen, Piao Guo, Yuchao He, Ziye Chen, Yi Luo, Liwei Chen, Lisha Qi, Xiaofang Zhang, Tianqiang Song, Hua Guo

Key: EA9C-553D-CB1B-B301-06AP

This certificate may be verified at **secure.authorservices.springernature.com/certificate/verify**.

Nature Research Editing Service is a service from Springer Nature, one of the world's leading research, educational and professional publishers. We have been a reliable provider of high-quality editing since 2008.

Nature Research Editing Service comprises a network of more than 900 language editors with a range of academic backgrounds. All our language editors are native English speakers and must meet strict selection criteria. We require that each editor has completed or is completing a Masters, Ph.D. or M.D. qualification, is affiliated with a top US university or research institute, and has undergone substantial editing training. To ensure we can meet the needs of researchers in a broad range of fields, we continually recruit editors to represent growing and new disciplines.

Uploaded manuscripts are reviewed by an editor with a relevant academic background. Our senior editors also quality-assess each edited manuscript before it is returned to the author to ensure that our high standards are maintained.
